# Trust the Process: A New Scientific Outlook on Psychodramatic Spontaneity Training

**DOI:** 10.3389/fpsyg.2018.02083

**Published:** 2018-11-14

**Authors:** Dani Yaniv

**Affiliations:** Emili Sagol Creative Arts Therapies Research Center, The Graduate School of Creative Arts Therapies, University of Haifa, Haifa, Israel

**Keywords:** spontaneity, psychodrama, bottom-up, top-down, creativity, transference, savant syndrome, role playing

## Abstract

Human mind is hypothesis-driven and our observations of the world are strongly shaped by preconceptions. This “top-down” principle is biologically driven and contraindicative to spontaneity, which is non-linear, condensed, and initially incomprehensible. My first argument is that spontaneity entails “bottom up” information processing, as articulated in the hierarchical neurocognitive model of perception. My second argument is that changing the balance between these two processes is important and feasible. Insights from psychodynamic transference and savant syndrome are presented to support these ideas. Uniting these contemporary notions with some essentials of J. L. Moreno’s philosophy is my third goal. By violating predictions and expectations, psychodrama interferes with top-down “conserved” processing and cultivates here and now, stimulus-dependent spontaneous acts. Further evidence is presented in support of the claim that adult spontaneity leads to enhanced cognition and creativity through imitating the child’s brain, as Moreno envisioned. Because spontaneity is formed before having the evidence for its truth or adequacy, it entails, in adults, overcoming apprehensions about acting without a theory in mind. This is what *trusting-the-process* means and it requires training, which psychodrama fosters on its stage laboratory.

## Introduction

“Man will fear spontaneity until he learns how to train it” (Moreno, 1953, p. 47).

*“Spontaneity is a function of organization”* (Wellman J. Warner, May 1951, as cited in *Moreno, 1953*, p. 545).

As a method of clinical intervention and group therapy, psychodrama uses a dramatic-theatrical format to allow clients enact emotions, experiences and meaningful events in life, thus turning the abstract into concrete. Through dramatic action, the client explores an internal world, reaching insights about self and others, experiences what could never happen, and develop better living skills. Based on action and enhancement of spontaneity and creativity, psychodrama assists clients in facing life challenges, examine alternative solutions, and sometimes adapt to their situation with peaceful acceptance. The protagonist in psychodrama is invited to *actually become* the “thing” that s/he is referring to, be it a person or an abstract concept, like passion. “The embodiment must correspond to the idea of the thing” (Moreno, 1946/1985, p. 26). This is enabled through spontaneous improvisation – a process that underpins psychodrama – and brings the patient closer to his/her emotions, thoughts, and imagination (Moreno, 1953).

Indeed, the origin of “spontaneous” is in Old Latin: [*sua*] *sponte*, “of one’s own accord, willingly” (dictionary.com. retrieved November 27th, 2017, from http://www.dictionary.com/browse/spontaneous?s=t). This definition emphasizes being in agreement and harmony with oneself, without external influence or constraint. Freedom is a central issue. Adjectives such as “automatic,” “impulsive,” or “instinctive” that sometimes accompany the term seem to underline a routine, mechanized and fixed patterned response, which, as elaborated below, seem to miss the point.

I argue that this unique psychodramatic experience corresponds to “bottom-up” processing (*vide infra*) and, accordingly, is particularly conducive to creativity training and therapeutic change. However, because we are inclined to think in a “top-down” fashion, spontaneity requires training, and the capacity to be trained and consequently change the balance exists in the general population. To begin substantiating this argument, I first refer to top-down vs. bottom-up processes as articulated in the cognitive neurosciences.

## The Hierarchical Model of Perception: “Bottom-Up” Vs. “Top-Down”

Classical theories considered the brain as passive and stimulus-driven, rather than as a device that actively creates meaning by itself. It was presumed to react to sensory inputs and copy pre-specified information. These approaches emphasized serial “bottom-up” processing in hierarchically organized neural structures (Marr, 1982; Biederman, 1987). Newer data designate the brain as much more active and adaptive (Edelman, 1989; Churchland et al., 1994; Engel et al., 2001). According to this view, cognition and behavior are not stimulus-driven, but are to a large degree based on expectations derived from previous experience and on generalized information already stored in the architecture of neural networks (Engel et al., 2001; Gilbert and Li, 2013; Muckli and Petro, 2013). This holds true in the infant brain as in adults (Emberson et al., 2015) and also when multisensory systems are involved (Lee Masson et al., 2016).

“Top-down” processing denotes cognitive influences and higher-order representations (e.g., expectations, attention, or knowledge) that impinge on earlier steps in information processing. “Bottom-up” refers to attention as driven mainly by the characteristics of the stimulus and its sensory context (e.g., contrast, symmetry, and order). The anatomical variant of this idea is that top-down influences are associated with the activity of descending pathways from the neocortex that are relayed through the thalamus, while bottom-up processes denote feedforward connections, ascending along a hierarchy of areas, which represent progressively more complex aspects of the sensory (visual) scene (Mumford, 1992; Ullman, 1995). Importantly, “top-down” and “bottom-up” processes represent general organizational principles rather than dichotomous concepts, and in most situations, they interact in the process of perception (Macaluso and Doricchi, 2013). However, the *proportion* or *dominance* of each of the processes within the interaction is dynamic and has important implications for cognition and behavior. Weighing of new (current) evidence and prior expectations must be dynamically adjusted when negotiating changeable real-world environments as well as clinical encounters.

Francisco Varela, a pioneer in embodied philosophy, suggested in 1987, that subjective visual perception is 80% dependent on ongoing “bussing activity” within the brain’s visual system, while only the remaining 20% is external and stimulus-dependent (Varela, 1987). Mahoney (1991) elaborated on this idea and suggested that the greatest proportion of that [internal] endless activity is self-referential (recursive):

Numerically speaking, there are 10 motor (efferent) neurons for every sensory (afferent) receptor; and for every motor neuron, there are 10,000 interneurons (neurons that connect only with other neurons). If we accept the traditional notion that one’s sensory receptors constitute one’s contact with the outside world, we are forced to conclude that one is much more extensively connected with oneself than with the external environment (pp. 101–102).

While controversy may still exist regarding the nature of perception (Firestone and Scholl, 2016), this principle (i.e., that most of our visual perception is internally driven) is biologically adaptive, as it promotes safety and avoidance of familiar painful experiences. Yet, it also induces a psychological/cultural burden, namely the difficulty to change. The fact that our mind is premise-driven and that our observations of the world are strongly shaped by pre-conceptions makes it difficult to embrace a new perspective (Gregory, 1980; Snyder et al., 2004). Chi and Snyder (2011) eloquently elaborate:

Information consistent with our expectations or mental templates is often accepted at face value, whereas inconsistent evidence is discounted or hidden from conscious awareness. While this hypothesis driven mechanism helps us in efficiently dealing with the familiar, it can prevent us from seeing better solutions in a different and/or unfamiliar context (p. 1).

I might add that it prevents us from finding better solutions in a *familiar* situation as well. For example, in a study on the Einstellung effect in chess, Bilalić et al. (2010) showed that even experts can fail to find an optimal solution when a common solution comes first to their mind. This demonstrates the powerful influence of our long-term memory and the strength that our preconceptions have on our mind (Gobet et al., 2014).

Furthermore:

Long-term memory is an important source of top-down processing, [it] includes not only declarative memories, but also the procedural knowledge stored in the functional architecture of sensorimotor networks. Network architecture could constitute an “implicit” source of top-down influences as, for instance, the topology of lateral connections within cortical areas is known to embody stored predictions that have been acquired both during evolution and through experience-dependent learning, and have proven to be of adaptive value (Engel et al., 2001, p. 714).

It appears, then, that the human mental apparatus has evolved to promote biological advantage by relying on existing knowledge more than on novel, unhabitual or unforeseen encounters (Varela et al., 1991/2016; Grossberg, 1999, 2000). To paraphrase Varela et al. (1991/2016), this quality is not optimal, it is, rather, simply viable. To be viable means that:

The perceptually guided action of the system must simply facilitate the continuing integrity of the system (ontogeny) and/or its lineage (phylogeny)…any action undertaken by the system is permitted as long as it is does not violate the constraint of having to maintain the integrity of the system and/or its lineage (Varela et al., 1991/2016, p. 205).

Therefore, the influence of ongoing top-down activity on the processing of incoming sensory signals is not confined to a feedback, but rather plays a decisive role in the (re)action production. Subjective information related to previous experience is superior in interpreting current perceptual stimuli, relative to the actual (bottom-up) input. Incoming signals may therefore convey different meanings about the same scene, in accord with a preexistent mental/behavioral context.

I suggest that intentionally changing the proportion of top-down/bottom-up dynamic processes, so that bottom-up input is more dominant at the expense of top-down influences, would enhance spontaneity as well as therapeutic change (*vide infra*). I also claim that this is exactly what psychodrama does, by cultivating spontaneity through role play and other creative acts. Before turning to the latter claim, I will substantiate the former using two clinical examples: psychological transference and autistic conditions. These examples demonstrate top-down and bottom-up dynamics in low-level sensory systems as well as in higher-order mental computations.

## Psychological *Transference*

Originally, within classical psychodynamic theory, transference was viewed as representing a displacement from the past, with the patient distorting the present in order to make room for the expression of some encapsulated earlier fantasy or experience. An alternative formulation sees transference as reflecting “a universal psychological striving to organize experience and construct meaning” that operates in an ongoing way, and which is “an expression of the continuing influence of organizing principles and imagery that crystallized out of the patient‘s early formative experiences” (Stolorow and Lachmann, 1984/1985, pp. 26, 25, as cited in Mitchell and Black, 1995, p. 166). Thus, transference distortions can and do exist in any interpersonal context, not only in therapist-client relationships^1^, and they are predominantly a top-down phenomenon in a context of attachment (Brockman, 1998, 2010). Transference triggers memory that “may enter consciousness and influence perception… where it takes control of attention, perception and thinking” (Brockman, 2010, p. 704). By focusing attention, transference “would be limiting perception—whether that focus be on perception from the senses or on perception from the medial temporal lobes (memory)” (ibid. p. 704).

Brockman (2010) further suggests that bottom-up *interventions*, in the context of psychodynamic treatment, consist of unexpected and unanticipated interventions and act as a “circuit breaker,” which leads to change. Violating expectations and predictions (as in “oddball paradigm”) would require a completely different course of action by the patient. Neurobiologically, this would be mediated through a ventral frontoparietal network that interrupts with a dorsal frontoparietal network that mediates top-down processing.

To illustrate, Brockman introduces a 48-year-old patient, who had been in numerous therapies since the age of 17. He accepted the referral from a colleague, who described the patient as “…chronically depressed, hopeless, suicidal” (p. 695). Among other reflections regarding this background, Brockman underlines: “I was partly aware that when I accepted this patient, it would be imperative that I find a treatment and a plan different from those she had undergone. I would need to find something new” (p. 695). After having reached a relatively solid therapeutic alliance, Brockman refers to a specific oddball intervention, in a moment when the patient complained about having a recursive failure in producing her work:

. . .

Therapist: Then maybe you should work here.

Patient: What?

Therapist: Here in my office. I’m not here many afternoons.

Patient: You’d let me work here?

(Therapist’s reflection): … I think we were both a little unsettled by the “oddball” quality of what

I had just offered. After a beat, she asked,

What makes you think there’s something so special about this office?

It’s not about the office. It’s about me, and your connection to me (p.708).

Instead of providing a “conventionally” therapeutic intervention, Brockman spontaneously said what could be considered the wrong sort of thing for a therapist to say. “What is spontaneity? It is the character of not resulting by law from something antecedent… I don’t know what you can make out of the meaning of spontaneity, but newness, freshness and diversity” (Peirce, 1935, p. 9, as cited by Moreno, 1946/1985). Within this particular therapeutic setting, Brockman seems to have reacted spontaneously while being genuinely empathic. Though somewhat unorthodox, similar examples appear in classical literature (Oremland, 1991) as well as self-psychology (Kohut, 1984).

Brockman clarifies that when transference work and interpretation are overpowered by the affective experience involved (through emotional memory), engaging in an unexpected and salient intervention shifts the balance, allowing “the prior focus to be released and a new focus initiated through the ventral system [to be] taken up as the new focus by the dorsal system” (Brockman, 2010, p. 706). Thus, within the transferential relationship, an unexpected and surprising suggestion created “a new object”^2^ and, as such, had to be perceived and processed through the bottom-up network.

## Insights From Autistic Savants

People with autism appear significantly less concept-driven than normal individuals (Snyder and Thomas, 1997; Snyder, 1998). While this lack of use of concepts leads to serious intellectual and social impairments, people with autism show exceptional performance on visual search tasks (Shah and Frith, 1983; Plaisted et al., 1998b; Joseph et al., 2009), have higher prevalence of absolute pitch (Miller, 1999), and superior visual discrimination (Plaisted et al., 1998a; Bertone et al., 2005), consistent with the development of positive neurology (Kapur et al., 2013; Schwarting and Busse, 2017). Particularly interesting, they are less susceptible to visual illusions than the normal population (Happé, 1996; Bogdashina, 2003).

Research suggests that people with autism see the world more accurately – as it really is – because they are less biased by previous experiences (Pellicano and Burr, 2012) or because of having a “privileged access to lower level, less processed information” (Snyder, 2009, p. 1399; see also: Frith and Happé, 1994). Recently, a third hypothesis was suggested, in which an increased propensity to represent and respond to environmental volatility (impaired top-down processing) compromises learning about probabilistic relationships in the environment, resulting in an increased receptiveness to sensory inputs (Lawson et al., 2017). In all of these potential explanations, people with autism are less susceptible to past-generated distortions/knowledge and are more open to alternative and, at times, more efficient and creative interpretations. This is especially evident in *savant syndrome* (from French *savoir* = to know), a specific and rare condition in which persons with autistic disorder or other mental disabilities have extraordinary skills, in stark contrast to their handicap (Treffert, 2009).

The condition can be present from birth or surface in early childhood (congenital) or can surface unexpectedly following head injury, stroke, dementia, or other central nervous system (CNS) disorders (acquired). The special skills occur most commonly in the areas of music, art, calendar calculating, lightning calculating, or mechanical/spatial abilities (Treffert and Rebedew, 2015, p. 158).

Snyder (2009) argues that savant skills are latent in all of us, an argument in accord with the fact that they can emerge “suddenly and spontaneously in individuals who had no prior history for them, either in interest, ability or talent” (p. 1400). Gobet et al. (2014) further elaborate and suggest that in the general population, “creativity can be boosted by decreasing conceptual processing and increasing the role of low-level perceptual processing” (p. 2). While in both autistic and healthy brains, creativity might be a separate process from talent or skill (Zaidel, 2014), Gobet et al. (2014) prediction was indeed tested and confirmed. Consistent with the view that autistic savants have some atypical anterior left brain dysfunction or inhibition together with right brain compensation (Miller et al., 1998; Treffert, 2005; Sacks, 2007), artificially inducing savant-like skills in normal healthy individuals using low-frequency repetitive transcranial magnetic stimulation (Snyder et al., 2006; Boggio et al., 2009; Snyder, 2009) or transcranial direct current stimulation (Chi and Snyder, 2012), brought a cortical disinhibition or atypical hemispheric imbalance; this in turn induced and improved savant-like abilities like drawing skills, proofreading skills, numerosity, and reduced false memories (for review see: Snyder and Mitchell, 1999; Snyder, 2009).

While the implications for the study of human cognition and the psychology of expertise are profound (Gobet et al., 2014; Wilson, 2016), it is enchanting to read a poetic description, so consistent with the above hypothesis, written as in 1953, early as by Moreno, father of psychodrama:

Spontaneity can enter the creatively endowed individual and evoke a response. There were many more Michelangelo’s born than the one who painted the great paintings, and many more Beethoven’s born than the one who wrote the great symphonies, and many more Christ’s born than the one who became Jesus of Nazareth. What they have in common are creativity and the creative ideas. What separates them is the spontaneity which, in the successful cases, enables the carrier to take full command of his resources, whereas the failures are at loss with all their treasures (p. 39).

Indeed, spontaneity (in contrast to impulsivity) is *the* cornerstone of psychodrama and is imperative in other creative arts therapies^3^ as well (Malchiodi, 2003). In some of the latter, however, *art* is essential to the therapeutic paradigm and is used mainly to project and thus to distance (zoom out) the person from his/her symbolic creation: visual arts through image, music through sound and rhythm, and poetry/writing through words (Knill et al., 1995). In psychodrama, the protagonist simply enacts his or her own life episodes, rather than, for example, some pre-given theatrical role, thus *zooming-in* without being significantly dependent on acting skills. While psychodramatic techniques for *zooming-out* are also used (e.g., “mirroring”), the basic method works through *nearing* to the psychic materials rather than distancing, and is the reason that psychodrama is considered a more direct method. Before further associating this to my argument, I will shortly introduce Moreno and psychodrama^4^.

## Some Basic Elements in Psychodrama

Moreno (1889–1974), a psychiatrist, who with his wife, Zerka Toeman Moreno, founded Psychodrama, which focuses on group processes and the primacy of action. Also a formidable pioneer of group psychotherapy and sociometry (the study and measurement of society and relationships), Moreno considered the three fields to be interrelated and indispensable to one another (Moreno, 1970). He had a strong existential and spiritual belief system, central to which was the treasuring of spontaneity, creativity, and the “urgency of immediate experience” (Moreno, 1989, p. 45, as cited in Wilson, 2011, p. 11).

In his Canon of Creativity, Moreno (1953) outlined a mutual association between spontaneity and creativity, so that the first arouses the second and the second is receptive to the first. “In order to become effective, creativity (*the sleeping beauty*) needs a catalyzer – spontaneity” (p. 45). It is generally accepted that Moreno’s intention was that spontaneity is a *state* that induce the creative sequence (*process*), leading to a final “product.” “Creativity is related to the “act” itself; spontaneity is related to the “readiness” of the act” (Moreno, 1955, p. 109). Without a doubt this twin concept was central in Moreno’s thought:

I formulated this twin concept as the primary principles of existence in my earliest effort to comprehend the living universe in its entirety. It seemed to me that they offer a safe bridge between ontology and science and that they are better able to explain all phenomena of the inanimate and animate universe than any other set of concepts I had known (ibid, p. 105).

The bridge, however, between ontology and science was not so safe, as Moreno’s definitions of spontaneity and creativity have been criticized for inconsistency (see: Aulicino, 1954; Kipper, 2000; Kipper et al., 2010). Is spontaneity an existential phenomenon, impossible to quantify or measure, as Bergson (1911) – frequently quoted by Moreno – suggested? Or is it a biologically based social phenomenon ready for empirical test? Kellermann (1992) reviewed this debate and suggested that despite Moreno’s natural science ideal, “in reality, Moreno’s humanistic bias shows through in most of his writing…[and] emphasize the hidden spiritual dimensions of reality and the intuitive, mystical sources of truth that cannot be investigated by the [objective] experimental approach” (p. 39).

In line with this suggestion, Moreno’s intention was to convince that spontaneity and creativity evoke levels of organized behavior which are not fully traceable to preceding determinants: “Whereas a living act is an element in the *causal-nexus* of the life process of the real person, the spontaneous-creative act makes it appear as if for one moment the *causal-nexus* has been broken or eliminated” (Moreno, 1946/1985, pp. 35–36). Relating this to a more clinical setting, Moreno conceived spontaneity important to treatment of mental disorders because it “enables the patient to… activate bodily and mentally his crucial conflicts so that he feels more clearly all the possibilities of a solution and eventually will turn his will towards a new path” (Moreno, 1939, p. 28).

Because spontaneity is not conservable like the kind of energies noted by physicists, and is vulnerable to mood, context, and mental dynamics, one has to *warm up* to it from the start. Indeed, “warming up” is formally the first phase in any psychodramatic meeting and stands for the activity of becoming gradually more spontaneous. It is indispensable in Moreno’s theory to get ready for action.

There are many ways to warm up to spontaneity, e.g., through physical action, promoting authentic encounters, or making abstract situations more concrete. Necessary to all is a safe and nurturing setting. Moreno was well aware of the fragility of this emotional process and of the spontaneous state itself. Yet he saw no other way but to rehearse, to train and to practice spontaneity through active, concrete, “experimental” actions, including role-playing. In *role play* one is deliberately creating an approximation of some aspects of a *real* experience, and it is through the study of roles *in action* that new knowledge about roles is developed and behavioral alternatives rehearsed (Moreno, 1953; Yardley-Matwiejczuk, 1997).

Indeed, among psychodrama’s most cherished techniques is *role reversal*: acting out one’s life roles and experimenting with new and unfamiliar (“other”) roles, with the help of an “auxiliary ego,” here and now, as if in their *statu nascendi* and *locus nascendi*. In a group context, selected auxiliaries usually enter some presented roles alternately, allowing an actual interaction to occur. Properly guided, such process is aimed at producing “a shift in *perception* so that one can see the other *and* oneself in a new and fresh way” (Kellermann, 1994; Moreno et al., 2000, p. 15). Hence,

Whereas conduct in a life situation is irrevocable, here every phase of the performance is open to correction through criticism made by the other participants, the instructor and the subject himself. Thus, a technique for learning to differentiate, in action, behavior patterns which may have been inadequate at the start is made available to the individual and to the group (Moreno, 1953, p. 534).

The *action* component, aptly led-to by a warm up, bypasses the rational, linguistic defensive mode of describing or explaining, allowing spontaneity and improvisation to arise (Moreno, 1953; Zwerling, 1979; Blatner, 2000). Using the instruction, “show me how” rather than “tell me why/what,” necessarily evokes unique neurological and psychological processes, critical to the success of therapy, that are missing when using discourse alone. A key concept in the construction of this notion is different types of conscious “selves,” with different inputs and qualities of consciousness (Markus and Wurf, 1987; Kahneman and Riis, 2005; Wilson, 2009; Conner and Barrett, 2012). Enactments naturally induce personal involvement by engaging an *experiencing self*, critically different from other selves’ states:

It is the experiencing self whose blood pressure rises in response to stressful situations (Kamarck et al., 2005), whose cortisol responds to a stressor (Smyth et al., 1998), and whose immune system reacts to elevated feelings of hostility during spousal fights (Kiecolt-Glaser et al., 2005). Although humans can evoke the stress response through memories and anticipated thinking (Sapolsky, 2004), acute autonomic, hormonal, and immune responses are most commonly activated as people act and react to life’s momentary stressors through the eyes of the experiencing self (Conner and Barrett, 2012, p. 6).

Cognitive processes induced during experiential action are predicted to differ considerably from those produced during rhetoric, static or non-experiential conditions (Yaniv, 2014a). Thus, acting from within or *acting out*, in psychodramatic practice, helps in gaining new insights and facilitates changing maladaptive behavioral patterns, which Moreno coined – “action insight” (Moreno, 1946/1985, p. x). “Acting out” in psychodrama means enacting rather than the psychoanalytic and more widely known usage that involves unconsciously expressing some disowned drive. As Moreno said,

I suggested that we differentiate two types of acting out, *irrational, incalculable acting out* in life itself, harmful to the patient or others, and therapeutic, controlled acting out, taking place within the treatment setting…under the guide of therapists who are able to utilize the experience (Moreno, 1946/1985, p. x).

In the latter, by “mixing” imagination with concreteness, truly creative acts become possible, invoke surprise and awe of the unexpected and add a new memory trace. On one hand, this enables the protagonist to process pain and mourn the past; on the other hand, it may allow experiencing compensation and correction. In total, this is an empowering experience that may change the protagonist’s point-of-view of himself and lead him further, to exercise creative behavioral plans, even in reality.

In the psychodramatic situation…the whole world into which the actor enters – the plots, the persons, the objects in it, in all its dimensions, and its time and space – are *novel* to him. Every step he makes forward in this world on the stage has to be defined anew. Every word he speaks is defined by the word which is spoken to him. Every movement he makes is defined, aroused and shaped by the persons and objects he encounters. Every step he makes is determined by the steps of others towards him. But their steps, too, are, at least in part, determined by his own steps (1946, p. 53).

Pushing it further, conceptually, Moreno envisioned psychodrama as having the potential to become a birth-like situation, with the protagonist becoming a new-born being:

The moment of birth is the maximum degree of warming up to the spontaneous act of being born into a new setting, to which he must make a rapid adjustment…the infant is the actor. He has to act in roles without having an ego or personality to act with. Like the impromptu actor, every step he makes in the world is new. He has to act quickly on the spur of the moment… (Moreno, 1946/1985, p. 54).

In contrast to this unique experience, Moreno’s concept of *cultural conserve* (Moreno, 1946/1985, pp. 107–109) is theoretically the opposite of spontaneity, insofar as it is a familiar, uncreative, and fixed form of engagement, but which is often required to set the stage for improvisation. Cultural conserves (e.g., the alphabet, the numbers, the language, and musical notations), underlie and determine all forms of creative activities. Moreno’s intention, when coining the term, was to refer to the category of things that already has been created, such as customs or social rules, in order to differentiate the *product* of creativity from the *process*, and to remind us to attend to the latter. He marked our “tendency to irrationally cling to what has been created, to rely on traditional or established rules as if they had unquestioned authority, to lapse into fixed or rigid habits of belief and thought” (Blatner, 2000, p. 75), instead of being spontaneous. Why does this tendency endure? “The answer is: man fears spontaneity, just like his ancestor in the jungle feared fire; he feared fire until he learned how to make it. Man will fear spontaneity until he learns how to train it” (Moreno, 1953, p. 47).

To summarize Moreno’s legacy regarding these issues, he saw the insufficient development of spontaneity as the source of human psycho- and socio-pathology. Therefore, he considered spontaneity training as “the most auspicious skill to be taught to therapists…and it is his task to teach his clients how to be more spontaneous without becoming excessive” (Moreno, 1953, p. 42).

In conclusion, the spontaneous-creative act and the cultural conserve are highly congruent with bottom-up and top-down processing, respectively: psychodrama encourages decreasing the “causal-nexus” inherent to top-down habitual behavior, and increasing “bottom-up” receptiveness by warming up to spontaneity, then creatively acting upon it. The latter is intensified in psychodrama, thanks to the association of personal content with dimensions of space, action, and imagination that are added to the more conventional verbal discussion in therapy. These dimensions “allow for improvisation, thinking in terms of alternative scenarios, shifting roles and points of view, opportunities for replay and other elements which offer new avenues to insight and self-reflection” (Blatner, 2000, p. xvi). Reaching these desirable goals requires considerable mental effort, to which I now turn.

## A Neurophenomenological Perspective

Whereas ordinary communicative processes (e.g., language) are perceived as known and linear, spontaneity (e.g., improvisational play) is non-linear, associational, condensed, and at times incomprehensible. In my view, *trusting the process* – a popular aphorism common in discussions of creativity in fields like arts, sports, and management – means remaining in the co-created improvisational (symbolic) play, despite its incomprehensibility. It requires overcoming the apprehension and nervousness aroused when working without a clear “theory” in mind. One has to willingly suspend control and become open, somewhat passive, “receptive” to stimuli and associations arising both within and without. This state reduces critique and judgmental (top-down) responses and “goes along” with whatever arises in the moment, even before understood. “The therapist can know whether his or her response is beneficial only by what happens next. The criteria chosen for this judgment are determined by the maturational purposes of the therapeutic endeavor” (Meares, 2001, p. 760).

From a neurophenomenological point of view, spontaneity is a disinhibition that allows a special kind of attention to the “thing in itself,” naked from expectations or preferences, reminiscent of the autistic savant experience. It has a relatively well-characterized brain profile, consistent with reduced frontal activation (Martindale, 1999; Yaniv, 2011, 2012, 2014a,b), which is also common in the “savant brain,” as discussed above. Does this imply that there are brain mechanisms common to psychodrama and savantism? As mentioned above, brain stimulation protocols that reversibly simulate savant lesions have been successfully used to induce creativity and enhanced cognition in normal subjects (Snyder et al., 2003; Young et al., 2004; Snyder et al., 2006; Boggio et al., 2009; Gallate et al., 2009; Karim et al., 2009; Chi and Snyder, 2012; Chrysikou et al., 2013). Gobet et al. (2014) suggest that these results give us a clue for “a ‘better’ brain— a brain that is hypothesis driven, but is resilient to cognitive illusions, a brain that can in addition see the world with direct perception and thus [be] open to alternative interpretations” (p. 1). Does the savant *kind* of knowledge, associated with bottom-up mechanisms, arise naturally in psychodrama?

Support of the suggested resemblance is based on the fact that the brain stimulation protocols mentioned above actually simulate the infant brain in which the frontal lobe matures gradually over the first years of life, in contrast to a much earlier maturation of other cortical areas. Thompson-Schill et al. (2009) review the late prefrontal development and suggest that despite some negative consequences for childhood behavior, it “has a clear advantage over an adult when it comes to certain types of learning (like language acquisition) or certain activities like flexible object use during problem solving” (p. 261). In fact, the authors review a few examples in which children – as well as patients with prefrontal cortex damage – do better than healthy adults in tasks that assess creative thinking. They further suggest that “This apparent flexibility of behavior can be interpreted as a stimulus-driven response: A mind that is at the mercy of its environment is not shaped by expectations or beliefs” (p. 262). The relation to psychodrama emerges through the move from the imagining of doing to actual doing, from hypothetical, imaginary forms of experimenting to real, witnessed and performed spontaneous acts.

Interestingly, the cradle of psychodrama was at children’s impromptu play at the gardens of Vienna around 1908–1911 (Moreno, 1946/1985, p. 3) and Moreno considered infants as “the geniuses of the race” (ibid. p. 48) from the perspective of embodiment and achievement. Much later, a few years after Moreno had moved to America, *The New York Times* published an interview with him entitled, “Impromptu plan used in education”:

Children, said Dr. Moreno in an interview, are endowed with the gift of spontaneous expression up to the age of 5, while they are still in an unconscious creative state, unhampered by the laws and customs laid down by preceding generations. After that they fall heir to accepted methods of expression; they become imitative, turn into automatons, and in a large measure are deprived of natural outlets of volitional creation (The New York Times, February 3, 1929; page number unavailable).

The remarkable association between Moreno’s early premises and recent scientific evidence, reasonably support the hypothesis that spontaneity, as regarded in psychodrama, is associated with hypofrontality and related compensatory-like mechanisms.

## So, What Comes of It: Bottom-Up, Top Down, or Both?

The dramatic incarnation of a stream of bottom-up experiences aroused in a protagonist is a professional asset of psychodramatists. It induces, in turn, non-habitual, creative, sometimes brave, impromptu actions in the psychodramatic scene. “It is not given like words or colors. It is not conserved or registered. The impromptu artist must warm-up, he must make it climbing up the hill” (Moreno, 1946/1985, p. 36).

Yet one has also to concentrate and self-organize in order to communicate – dramatically or otherwise – whatever arises, which may be blurred, even distorted. The two phases continuously interact during the spontaneous-creative act, reminiscent of the double-phased “regression in service of the ego” (Kris, 1936/1952) which enables the maintaining of contact with primary physical and mental positions and with varied thinking modes (Knafo, 2002). This complexity was eloquently described by Moreno:

In the spontaneous-creative enactment, emotions, thoughts, processes, sentences, pauses, gestures, movements etc., seem first to break formlessly and in anarchistic fashion into an ordered environment and settled consciousness. But in the course of their development it becomes clear that they belong together like the tones of a melody; that they are in relation similar to the cells of a new organism. The disorder is only an outer appearance; inwardly there is a consistent driving force, a plastic ability, the urge to assume a definite form (Moreno, 1946/1985, p. 36).

While the move from the “anarchistic fashion” to “an ordered and settled consciousness” *could* be understood as a move in dominance of mental processes – from bottom-up to top-down, I do not think that this is the case. This move is a process that creates a new insight, and thus it cannot be identified as a top-down (cultural conserved) phenomenon, but rather a rearrangement of current mental representations and therefore continuous in nature with the preceding spontaneous-creative state of mind. This distinction is important in understanding spontaneity the way Moreno conceived it:

One of the contributions of spontaneity research was to recognize the various phases and degrees of spontaneity as one continuous process, the reduction and loss of spontaneity, impulsive abreaction and the pathological excesses as well as adequate and disciplined spontaneity, productive and creative spontaneity. Another contribution was to recognize that spontaneity does not operate in a vacuum but in relation to already structured phenomena, cultural and social conserves. Spontaneity is a function of organization (1953, p. 545).

Eventually, this process may be consolidated into long-term memory, becoming the new top-down, conserved baseline. This may be best represented in Moreno (1946/1953) original concept – “catharsis of integration.” He considered catharsis as:

A process which accompanies every type of learning, not only a finding of resolution from conflict, but also a realization of self, not only release and relief but also equilibrium and peace. It is not a catharsis of abreaction but a *catharsis of integration* (1953, p. 546).

Moreno believed that as a *consequence* of role reversal, the actor-patient has an opportunity to find and re-organize him/herself, “to put the elements together which may have been kept apart by insidious forces, to integrate them and to attain a sense of power and of relief, a catharsis of integration (in difference from a catharsis of abreaction)” (1953, p. 85). It can well be said that through a long chain of role takings and interactions, dialogical sequences and pauses, moments of meditation and decision, psychodrama aims at an integration of the protagonist with his co-actors and the spectators of the drama, into a freer and more spontaneous community, in the group microcosmos and beyond. As a result, psychodrama must be considered as first and foremost a bottom-up method and process. By violating predictions and expectations, psychodrama interferes with top-down processing and cultivates in-the-moment, stimulus-dependent experiences, from beginning to end.

## Limitations and Conclusion

While the present arguments clearly point to a past-dependent constriction of cognition, it is only fair to note that a contrasting view had been previously presented, whereby top-down processing allows the agent *freedom from immediacy* (Shadlen and Gold, 2004) by not being “limited to that which emerges as a direct response to stimulus input” (Smallwood et al., 2013, p. 120). Designating attention to the immediate environment as *freedom or captivity* seems beyond experimental considerations and may consist of personal preferences which are beyond the scope of the present paper. Suffice it to mention the central role attention to immediacy is given in the Zen/Buddhist meditation practice and in its modern counterpart, mindfulness meditation. The relationship of the latter to drama-related therapies is innovative and has been recently discussed elsewhere (see: Gluck, 2013; Schuchner, 2016; Yaniv and Kedem, 2017).

A more objective challenge emerges regarding the lack of empirical consensus about the neuroscience of creativity (Arden et al., 2010; Dietrich and Kanso, 2010; Sawyer, 2011), raising questions such as what is the relative importance of top-down vs. bottom-up processing in creativity (Thompson-Schill et al., 2009; Benedek et al., 2011), or whether creativity can be isolated to discrete regions in the brain (Abraham, 2013; Jung et al., 2013). A likely explanation for these inconsistencies is that results from individual studies are framed in general terms, while neither creativity nor spontaneity should be treated as a single entity. For example, when looking at individual components of general creative ability (e.g., divergent thinking), an emerging literature has yielded a relatively consistent pattern of results, pointing to the importance of functional connectivity between different brain areas (Beaty et al., 2014, 2016).

Introducing the bottom-up/top-down conceptualization to psychodramatists is new and important because it may provide them with a different perspective on their actual interventions, thus opening new possibilities for developing psychodramatic theory and practice, and it could recast psychodrama in a more scientific light. On the other hand, introducing spontaneity – from the psychodramatic perspective – to neurocognitive science could prove beneficial, as it could show how the fast, spontaneous, unknown side of our mind can bring about appropriate, competent, and skillful responses “in dealing with a situation, however, small or great the challenge of its novelty” (Moreno, 1955, p. 109). It might also open up a rich array of in-action experimental procedures that are currently lacking in the field (Yaniv, 2014a). For example, in a study about divergent thinking, *openness to experience* was conceptualized as a “tendency to engage in imaginative, creative and abstract cognitive processes” (Beaty et al., 2016, p. 773) and was evaluated using scale questionnaires. From a spontaneous point of view, openness to experience would be authentically appraised by an actual engagement in some improvisational, creative *acts*. Using *this* kind of research tool would make the stemming results more valuable for real life situations and clinical applications (Cole, 2001; Nadar and McDowd, 2008; Beidas et al., 2014; Yaniv, 2014a). Psychodrama has been successfully implementing this vision for over a century. Other clinical traditions, like contemporary psychoanalytic psychotherapy, began incorporating some of these unique characteristics more recently (Rothenberg, 1988; Modell, 1997, 2009; Stern, 2007; Wachtel, 2009). The challenge to the cognitive sciences remains.

## Author Contributions

The author confirms being the sole contributor of this work and has approved it for publication.

## Conflict of Interest Statement

The author declares that the research was conducted in the absence of any commercial or financial relationships that could be construed as a potential conflict of interest.

## Footnotes

1 However, the therapist-client relationship incorporates a regressive component, which tends to increase the stress of the relationship and consequently increases the potential for transferential distortions.

2 This, of course, may contribute therapeutically by leading toward a transformation of the therapist from being an “object” to being a subject.

3 Conventionally including art, music, drama, dance/movement, poetry/creative writing, bibliotherapy, play, and sandplay.

4 The philosophical and theoretical basis of classical psychodrama is voluminous, and only few selected elements are tapped here. The interested reader is advised to refer to: Moreno (1946/1985, 1953), Moreno and Moreno (1956, 1975), Moreno et al. (2000), Horvatin and Schreiber (2006), von Ameln and Wieser (2014).
